# Treatment of selective mutism: a 5-year follow-up study

**DOI:** 10.1007/s00787-018-1110-7

**Published:** 2018-01-22

**Authors:** Beate Oerbeck, Kristin Romvig Overgaard, Murray B. Stein, Are Hugo Pripp, Hanne Kristensen

**Affiliations:** 1 Department of Mental Health and Addiction, Oslo University Hospital, Nydalen, Po box 4959, 0424 Oslo, Norway; 2 University of California, San Diego, La Jolla, CA USA; 3 Department of Biostatistics, Epidemiology and Health Economics, Oslo University Hospital, Oslo, Norway; 4 Centre for Child and Adolescent Mental Health, Eastern and Southern Norway, Oslo, Norway

**Keywords:** Selective mutism, Child anxiety, CBT, Quality of life, Self-report

## Abstract

Selective mutism (SM) has been defined as an anxiety disorder in the diagnostic and statistical manual of mental disorders (DSM-5). Cognitive behavioral therapy (CBT) is the recommended approach for SM, but prospective long-term outcome studies are lacking. Reports from the children themselves, and the use of more global quality of life measures, are also missing in the literature. We have developed a school-based CBT intervention previously found to increase speech in a pilot efficacy study and a randomized controlled treatment study. Continued progress was found in our 1-year follow-up studies, where older age and more severe SM had a significant negative effect upon outcome. In the present study, we provide 5-year outcome data for 30 of these 32 children with SM who completed the same CBT for mean 21 weeks (sd 5, range 8–24) at mean age 6 years (10 boys). Mean age at the 5-year follow-up was 11 years (range 8–14). Outcome measures were diagnostic status, the teacher- and parent-rated selective mutism questionnaires, and child rated quality of life and speaking behavior. At the 5-year follow-up, 21 children were in full remission, five were in partial remission and four fulfilled diagnostic criteria for SM. Seven children (23%) fulfilled criteria for social phobia, and separation anxiety disorder, specific phobia and/or enuresis nocturna were found in a total of five children (17%). Older age and severity at baseline and familial SM were significant negative predictors of outcome. Treatment gains were maintained on the teacher- and parent questionnaires. The children rated their overall quality of life as good. Although most of them talked outside of home, 50% still experienced it as somewhat challenging. These results point to the long-term effectiveness of CBT for SM, but also highlight the need to develop more effective interventions for the subset of children with persistent symptoms.

*Clinical trials registration* NCT01002196

## Introduction

Selective mutism (SM) is characterized by a consistent lack of speech in specific social situations in which there is an expectation for speaking (e.g., school) despite speaking in other situations (e.g., at home) [1, 2]. SM is relatively rare, with a prevalence of about 1% in childhood, somewhat more frequent in girls [3] and bilinguals [4] and age of onset is typically before age 5 years [5–7]. SM has been found to co-occur with other anxiety diagnoses (particularly social phobia) and with neurodevelopmental disorders [7–12]. SM is also reported to run in families, and a family history study of 38 children with SM reported a clear excess of the personality trait of taciturnity in 1st-, 2nd-, and 3rd-degree relatives, underlining the importance of the familial background for outcome studies [13]. Support for a familial relationship between social phobia and SM was found in parents to children with SM [14]. Due to the gradual shift in the understanding of SM from an act of will to an anxiety-based avoidance of speaking in specific situations, SM was classified as an anxiety disorder in the fifth edition of Diagnostic and statistical manual of mental disorders (DSM-5) [2].

SM has over the years been considered difficult to treat, and both medication and psychosocial treatment have been tried. Concerning medication, a systematic review found some evidence for symptomatic improvement in SM with selective serotonin reuptake inhibitors (SSRIs) [15]. However, controlled- and naturalistic follow-up studies have noted that both treated and untreated children were still very symptomatic, and/or diagnoses persisted [16, 17].

The psychosocial treatment literature for SM was long dominated by case studies or case series with quite diverse treatment approaches. A comprehensive practitioner review from 2006 provided support for the use of behavioral- and cognitive behavioral therapy (CBT) for SM [18]. In the following years, different CBT approaches have been reported to lead to symptom improvement in case series using both individual- and group formats [19, 20].

In the literature, data are still scarce on both long-term outcome and predictors of outcome. Using retrospective patient records, persisting communication problems were found in a substantial portion of 45 children with SM at mean 12 years after treatment, while 39% were considered to be in full remission [21]. Furthermore, the study found that a poor outcome was best predicted by mutism within the core family. In another study, although SM improved (57%), a high rate of psychiatric disorders (57%) was found in 33 adults (61% females) with a childhood SM diagnosis. The phobic disorders (including social phobia) were the most frequent diagnoses (42%), and the study found that a severity indicator of SM, taciturnity in the family, and, by trend, immigrant status had a negative impact on psychopathology and symptomatic outcome in young adulthood [22]. In 25 children, those given individual programs with a behavioral component were more likely to have improved compared with those given standard school-based remedial programs, 2–10 years after referral, and familial psychopathology was a negative prognostic factor [23].

In recent years, CBT interventions especially adapted for children with SM have been elaborated. The behavioral components have been emphasized, as the symptom of muteness and the young age of onset of SM make the cognitive restructuring less feasible. As children with SM tend to be most symptomatic at school [24], extensive cooperation with teachers is required. Furthermore, a special strategy to secure early child engagement, as well as parental involvement is vital, as children with SM often fail to speak to the therapist. Consequently, in 2013 researchers developed an integrated behavioral therapy for SM [25] to be conducted at the clinic with parental participation using graduated exposure tasks to the feared stimuli/situation (e.g., verbal communication). When appropriate to the developmental level of the child, selected cognitive restructuring principles were used (e.g., replacing fearful or worried thoughts with coping self-statements). A pilot randomized controlled study (RCT) [26] including 21 children (4–8 years of age) found a significant increase of speech after treatment, with no change in wait-list controls, as rated by the teachers on the school speech questionnaire (SSQ) [3]. Furthermore, 67% of treatment recipients were not considered to fulfill criteria for SM, and clinical gains were maintained at 3-month follow-up [26]. In 2016, a retrospective naturalistic study examined the outcome in 24 of 36 children with SM (mean age 6 years) who had been treated for mean 12 months with a specially designed modular cognitive behavioral therapy (MCBT) by one therapist at one clinic [27]. In addition to the behavioral interventions (contingency management, graded exposures tasks, modeling and shaping), relaxation training, and psychoeducation (including training of parents and educational staff on how to facilitate speech), the study used cognitive training (externalizing the symptoms and cognitive restructuring). The outcome, mean 3 years after end of treatment was highly favorable, as 84% of the children had recovered from SM.

In 2017, a pilot study examined the outcome of another multimodal approach (the Social Communication Anxiety Treatment; S-CAT) in 33 of 40 children with SM [28]. They were treated at mean age 6 years for mean 9 weeks by one therapist at a private specialty practice for SM. In addition to psychoeducation and graded exposure tasks essential in CBT for SM, this approach uses an extensive transfer of control to the parents already at the first therapy session. Parents were taught how to implement social communication goals by taking activities from therapy sessions into public places by the therapist, who also closely monitored their compliance. This pilot study did not use diagnoses. Significant gains were found on parent severity ratings (the selective mutism questionnaire; SMQ), at mean 6 weeks after end of therapy, with more severe SM and noncompliance from parents as negative predictors of outcome.

In contrast to the above mentioned clinic-based treatments, our intervention is a school-based CBT, as children with SM tend to be most symptomatic in this environment [24]. To promote rapport with the child, increase parental engagement, and train on procedures later to be used at preschool/school, the treatment started at home (three sessions), where these children feel most safe. To decrease the often co-occurring social anxiety, we used defocused communication as a general treatment principle. The central components are: To sit beside rather than opposite the child; to create joint attention using an activity the child enjoys rather than focusing on the child; to ‘think aloud’ rather than asking the child direct questions; to give the child enough time to respond rather than talking for the child, to continue the dialog even though the child does not respond verbally; and try to receive a verbal answer in a neutral way rather than praising the child.

In line with the practitioner review recommendation by Cohan et al. [18], and the consensus based care pathway of good practice by Keen et al. [29], we chose to use psychoeducation and behavioral interventions. The psychoeducation (including information about SM, and how to use defocused communication) was given by phone with the teachers and parents together to obtain a mutual understanding of SM and the child’s level of functioning. The behavioral interventions consisted of stimulus fading in the form of gradual increased exposure, as well as contingency management (use of positive reinforcement for speaking behavior) to be applied in a joyful play activity inspired by the Selective Mutism Resource Manual [30]. The behavioral interventions took place at preschool/school twice a week (each lasting half an hour) and followed six defined modules/speaking levels according to the progress of the child. The parents participated in the first module, the teachers from modules III to VI and peers/classmates from modules IV to VI (a more thorough description of the modules and the intervention is available in the RCT study [31]). Due to the feasibility of the study, the maximum length of treatment was set to 6 months. The treatment was discontinued if the child started to speak freely before reaching the maximum length of treatment (6 months).

Using this school-based CBT, we found a highly favorable treatment outcome in a pilot efficacy study of seven preschool children with longstanding SM [32]. All but one child spoke freely in all preschool settings after a mean of 17 weeks treatment (sd 5, range 8–24 weeks).

At follow-up 1 year after end of treatment, this child had SM in partial remission; the others did not have SM. Using the SM questionnaires, two children showed a transient drop of scores related to their transition into school, while treatment gains were upheld in the others. Bilingual children comprised the majority in this study, suggesting that bilingualism may not be a central negative outcome predictor.

We also found a significant treatment effect in an RCT study of 24 children with SM, 3–9 years of age, with no change in wait-list controls, using this school-based CBT applied by local therapists at community health clinics all over Southern Norway [31]. In the RCT study, where all had a principal diagnosis of SM and comorbid social phobia, and 2/3 had additional diagnoses, the children were randomized to 3 months of treatment or wait-list controls. A time by age interaction favored younger subjects. After 3 months, the children in the waitlist group received the same treatment. In this effectiveness study, there was a significant increase of speech after mean 23 weeks of treatment (sd 3, range 12–24 weeks), with continued progress measured 1 year after the end of treatment using the teacher-rated SSQ and diagnostic status as primary outcome measures [33]. There was one treatment drop-out after 3 months in this effectiveness study, but all children had complete outcome data. While older age and more severe SM at baseline had a significant negative effect upon outcome, we found no significant effect of gender or familial SM. In this study, all but one family reported that mother and/or father had childhood social anxiety, and SM had been present in other family members in 10 of the 24 families (42%).

As SM is defined as an anxiety disorder, the literature on pediatric anxiety disorders is relevant for the present study. Pediatric anxiety disorders can be effectively treated in the short term, and predictors of remission were found to be younger age, nonminority status, lower baseline anxiety severity, absence of other internalizing disorders, and absence of social phobia [34]. The overrepresentation of bilinguals, and the high proportion of comorbid anxiety disorders, especially social phobia in children with SM [7], makes the literature on pediatric anxiety disorders particularly relevant for SM outcome studies and could suggest a poorer outcome.

Data are limited on the long-term outcomes of pediatric anxiety disorders. The important Child/Adolescent Anxiety Multimodal Extended Long-term Study (CAMELS) found that relapse occurred in almost half (48%) of acute responders when assessed at mean 6 years after randomization [35]. A systematic Cochrane review stated in 2015 that the few controlled follow-up studies (*n* = 4) indicate that treatment gains in the remission of anxiety diagnosis are not statistically significant [36].

Over the years, a subjective perception of well-being has been recognized as an important complement to clinical symptomatology and functional impairment in CAMHS. Quality of life measures could be one way of giving weight to the child’s perspective. For children with SM, findings on quality of life, as well as the use of children as informants are missing in the outcome literature. A systematic review on how childhood mental disorders affect quality of life in general conclude with a significant reduction compared to healthy controls across several disorders, and that studies for large diagnostic groups (for instance anxiety disorders), are largely lacking [37].

The aim of the present study was to expand the literature on the anxiety disorder SM in two ways: by providing prospective long-term outcome data in a relatively large number of children with SM who completed the same school-based CBT and by including data on child rated quality of life and their own speaking behavior.

We report data on 32 children, from our pilot study (*n* = 7), our RCT study (*n* = 24), and one child with SM not included in the RCT study due to being a sibling of a child in the RCT.

Based on the existing literature on pediatric anxiety disorders, where factors such as bilingualism, and comorbid anxiety disorders, especially social phobia are found to be negative predictors of treatment outcome [34] one could hypothesize a poor long-term outcome in children with SM, where these factors are overrepresented [7].

However, based on the favorable results in our previous follow-up studies conducted 1 year after end of treatment [32, 33], we hypothesized that treatment gains would be maintained in the present 5-year follow-up study.

## Method

### Design

This is a prospective long term follow-up study conducted at mean 5 years after the end of our school-based CBT especially adapted for children with SM. Data include the present follow-up (T5), as well as from baseline (T1), after 3 (T2) and 6 (T3) months of treatment, and 1 year after end of treatment (T4).

#### Participants

The sample consists of 30 of the total 32 children who completed a school-based CBT for SM in Norway, seven children from our pilot study [32] and 24 children from our RCT [31] and one child not included in the RCT who received the same treatment by one of the therapists in the study. Mean age at inclusion was 6 years (range 3–9 years) and mean age at follow-up was 11 years (range 8–14), including 20 girls, and 9 bilingual children. Familial SM was found in 11 of the 30 participating families (in parents: *n* = 4, in parents and siblings: *n* = 3 or in grandparents or aunts/uncles *n* = 4). The two non-participating families did not reply to our follow-up invitation, and both children had SM and social phobia when assessed at the 1-year follow-up study [33].

#### Baseline inclusion criteria

Children aged 3–9 years, consecutively referred from outpatient Child and Adolescent Mental Health Clinics (CAMHS) or school psychology services in Southern Norway who fulfilled DSM diagnostic criteria for SM. In addition, we specified that the children should not speak to adults in preschool/school, and that mutism was present in both languages for bilingual children. Our rationale for the operationalizing of SM as not talking to teachers was that a detailed description is missing in the diagnostic criteria. By giving a description of how we defined SM we could allow for study replication.

#### Baseline exclusion criteria

(1) Parents who did not speak Norwegian or (2) children with IQ < 50, psychosis or a Pervasive Developmental Disorder. The final inclusion was confirmation of the SM diagnosis after a parental diagnostic interview and a child assessment to rule out severe intellectual problems. At baseline, nonverbal IQ and receptive language were within the average range [31, 32].

#### Treatment

All children were treated with our school-based CBT (the treatment is described in the introduction) by local clinically experienced therapists who had no (*n* = 4), some (*n* = 11), or extensive (*n* = 6) previous work with selectively mute children. None had specific CBT training, but used our detailed manual describing defocused communication and weekly behavioral school-based interventions for a maximum of 6 months (mean 21 weeks, sd 5, range 8–24) under supervision from the first or last author, with no further treatment adherence measures.

### Assessment instruments

Outcome measures were diagnostic status, the teacher- and parent-rated selective mutism questionnaires, and child rated quality of life and speaking behavior. See Table 1 for an overview of measures and informants at T1 through to T5.

**Table 1:** Overview of informants and measures throughout the study, at baseline (T1), after 3 months of treatment (T2) end of treatment; 6 months (T3), 1 year after end of treatment (T4) and after 5 years (T5)

Informants	Time points for data collection				
	T1	T2	T3	T4	T5
Teacher	SSQ	SSQ	SSQ	SSQ	SSQ
Mother	SMQ	SMQ	SMQ	SMQ	SMQ
Mother	ADIS-IV; SM module K-SADS-PL			ADIS-IV; SM module K-SADS-PL	ADIS-IV; SM-module K-SADS-PL
Child					ILC
Child					One speaking item^a^

*SSQ* school speech questionnaire, *ADIS* anxiety disorders interview schedule (ADIS-IV), *K*-*SADS*-*PL* schedule for affective disorders and schizophrenia for school-aged children: present and lifetime version, *SMQ* selective mutism questionnaire, *ILC* the inventory of life quality in children and adolescents

^a^The item is scored 1–5; equivalent to the ILC

### Diagnosis of SM and comorbid diagnoses

SM was diagnosed using the SM module from the semi-structured Anxiety Disorders Interview Schedule (ADIS-IV) [38] with good construct validity [39]. The SM module relates to the speaking behavior of the child in different social situations. We chose to use three categories of SM:

1. Full remission: children who no longer fulfilled diagnostic criteria for SM, as they spoke freely at school.
2. Partial remission: children who spoke freely in some, but not all settings at school. (E.g., often, but not always when being approached by a teacher in the classroom, or to a teacher when they were in a small group of students). Thus, rigorously speaking they did not fulfill the DSM criteria of “Consistent lack of speech”.
3. SM: children who fulfilled diagnostic criteria for SM.

To assess diagnostic comorbidity, we used the revised version of the schedule for affective disorders and schizophrenia for school-aged children: present and lifetime version (K-SADS-PL) [40]. The second author, an experienced child psychiatrist, conducted the interviews, blind to diagnostic status. Interrater reliability was assessed by rescoring the audiotapes resulting in a 100% agreement on whether SM was absent or present/in remission, and good agreement (kappa > 0.70) for the other anxiety disorders. The parents were also asked whether they had been in contact with CAMHS or the school psychology services during the follow-up period.

### SM questionnaires

#### The school speech questionnaire (SSQ) [3]

The SSQ is based on speech frequency in the school context and was rated by the child’s teacher at T1 through to T5. It is a quantitative measure with no cut-off score, includes 10 questions modified from the SMQ (see below) with acceptable internal consistency. Six of the SSQ questions (identical to the SMQ) are used to compute a mean score (range 0–3). As in the SMQ, 0 indicates that speaking behavior never occurs, and 1, 2, and 3 refer to seldom, often and always speaking, respectively. In the present study we used the Norwegian translation, available at http://iacapap.org/wp-content/uploads/F.5-MUTISM-NORWEGIAN-2016.pdf with good internal consistency (*α* = 0.84).

#### The selective mutism questionnaire (SMQ) [24]

The SMQ was rated by mothers at the same time points (T1–T5). The SMQ includes 32 questions scored from 0 to 3, where 0 indicates that speaking behavior never occurs, and 1, 2 and 3 refer to seldom, often and always speaking, respectively. Seventeen of the SMQ questions are used to compute three subscale mean scores; at school (six items), at home (six items) and in public (five items) with the same 0–3 scoring range, computed as the mean of the relevant items. The SMQ total factor score was computed from the sum of three subscales divided by three. In the present study we used the Norwegian translation, available at http://iacapap.org/wp-content/uploads/F.5-MUTISM-NORWEGIAN-2016.pdf with acceptable to excellent internal consistency on the three subscales and the total score, respectively (*α* = 0.82, 0.76, 0.90, 0.93).

The SM questionnaires are quantitative measures with no cut-off scores, but a psychometric SMQ-study suggested a score ≤ 0.5 on the School subscale for children with SM, and ≥ 2.5 for those without SM made after examination of only *n* = 18 children with anxiety disorders other than SM [24].

### Child rated inventory of life quality

The Norwegian version of the German Inventory of Life Quality in Children and Adolescents (ILC) was used [41, 42]. ILC consists of seven items. Six items address subjective well-being at school, in the family, with peers, when alone, and perception of physical and mental health with a final global item of life quality. Each item is rated on a 1–5 scale (1 = very good through 3 = mixed, to 5 = very bad). A review on the published studies on the Norwegian version of the ILC concluded that although there is limited documentation for the psychometric properties of the Norwegian ILC, the existing four studies are of good quality, including satisfactory norms and measures of validity and reliability [43].

We report three ILC scores:

I. The ILC Life Quality score (LQ_0–28_) calculated by multiplying the mean of the seven items by seven. The scores on the 1–5 scale are reversed so that a LQ_0–28_ value of zero indicates very low LQ and 28 very high LQ, and the general rule of interpretation is that score < 15 suggests a life quality below the mean. Mean LQ_0–28_ score 22.59 (sd 3.88) was reported in Norwegian school children aged 8–16 years (*n* = 1987) [41].
II. Mean ILC subscale scores, using the 1–5 ratings on the individual subscales (normative data not available).
III. The ILC problem score (PR) computed by dichotomizing each of the seven subscales, such that ratings of 1 or 2 indicates no problem (0), and ratings of 3, 4 or 5 indicates that a problem is present (1) on the subscale. A mean ILC problem score can then be calculated (range 0–7), where a score of 1.28 (sd 1.60) has been reported in Norwegian school children aged 8–16 years (*n* = 1987) [41]. Normative Norwegian data are also available for the percentage of problems per subscale (presented as a comparison in Fig. 2) [44].

### Child ratings of their own speaking behavior

Standardized SM questionnaires for children are not available. To obtain some form of child report we asked the children to rate their difficulties with speaking at school/outside home on a Likert scale, corresponding to the 1–5 range on the ILC, especially adapted by the authors for the present study (1 = very easy, through 3 = mixed), to 5 = very difficult). A mean score range (range 1–5) and a problem score (PR) (where ratings of 1 or 2 indicates no problem, and ratings of 3, 4 or 5 indicates that a problem with speaking is present), was computed.

Child ratings were available from 28 of the participating 30 children.

### Ethical approval

Written informed consent was provided by the parents and children (from age 11 years). The study was granted approval by the Norwegian Social Science Data Services and the Regional Committees for Medical and Health Research Ethics.

### Data analysis

Descriptive statistics using mean/standard deviation (sd) or number/percentage of patients are presented for the diagnoses, SM questionnaires (SSQ, SMQ), the ILC and speaking behavior. A linear mixed model for repeated measurements using a random intercept for each subject was applied to investigate the SM questionnaires scores from baseline (T1), 3 months (T2), 6 months (T3), 1 year after end of treatment (T4) and 5-year follow-up (T5). A fixed effect of age group at diagnosis (3–5 years versus 6–9 years) and a time × age group interaction were examined in an additional analysis. Post hoc analysis of mean differences between the five time points (T1–T5) were tested using Bonferroni corrections. The level of significance was defined as *p* < 0.05. A Chi-square test was used to calculate the differences in comorbid disorders between T4 and T5, the difference between boys and girls, and between children who were/were not bilingual, and had/had not SM in the family. An independent samples *t* test was conducted to calculate the difference in Quality of life scores between the children in the present study and Norwegian schoolchildren. A one-way ANOVA with Tukey post hoc comparisons was used to test for differences in the SM questionnaires at follow-up (T5) between the three SM groups (No Sm, SM in partial remission, SM), and for differences in baseline severity at T1 as assessed by the SMQ between the three SM groups (No Sm, SM in partial remission, SM at follow-up (T5).

## Results

### Diagnostic status

At the 5-year follow-up, 70% of the children (*n* = 21) no longer fulfilled diagnostic criteria for SM, as they spoke freely at school and were considered in full remission. Another 17% (*n* = 5) spoke freely in some, but not all settings at school and were categorized as SM in partial remission. The remaining 13% (*n* = 4) continued to fulfill diagnostic criteria for SM.

When investigating the different individual courses of development, most of the children showed continuous progress, but three children changed status negatively. One school-age child had a relapse of SM after having SM in partial remission at the 1-year follow-up. Two children (one preschool- and one school-age child) who at the 5 year follow-up did not speak in all school situations were diagnosed with SM in partial remission, after having been fluent speakers at the 1-year follow-up.

Based on the diagnostic categories of SM, we found a more prominent improvement in the younger children, as 14 of the 16 children (88%) aged 3–5 years at inclusion were in full remission at the 5-year follow-up, compared with seven of the 14 children (50%), aged 6–9 years at inclusion (*χ*^2^ square 4.99, *df* = 1, *p* = 0.03).

Furthermore, baseline severity as assessed by the SMQ total score showed a significant negative impact upon outcome (*F*_2,29_ = 4.41, *p* = 0.05), and the pair of groups found to be statically significant was SM versus SM in full remission (*p* = 0.05).

Among the eleven children with SM in the family, two children had SM and four children were diagnosed with SM in partial remission. Only five of these 11 children (45%) did not fulfill diagnostic criteria for SM, significantly fewer than among children without SM in the family, where 17 of 19 children (89%) did not have SM (*χ*^2^ 12.92, *df* = 1, *p* < 0.001). Among the twelve bilingual children, eight (67%) did not have SM, not significantly different from the 13 of the 18 monolingual children (72%) (*χ*^2^ 0.17, *df* = 1, *p* = 0.68). Six of the ten boys (60%) and 15 of the 20 girls (75%) did not have SM at follow-up, the difference was not significant (*χ*^2^ 0.69, *df* = 1, *p* = 0.41).

Comorbid diagnoses, as assessed by K-SADS, revealed that 23% (*n* = 7) fulfilled criteria for social phobia, significantly fewer than 79% at the 1-year follow-up, *χ*^2^ 16.45, *df* = 1, *p* < 0.001 (at baseline the rate was 100%). Among these seven children, only two did not also have SM, or SM in partial remission. Additional diagnoses, other than SM and social phobia, were separation anxiety disorder (*n* = 2), specific phobia (*n* = 3) and enuresis nocturna (*n* = 1) in a total of five children (17%), significantly fewer than 50% at the 1-year follow-up (*χ*^2^ 6.59, *df* = 1, *p* = 0.01) (at baseline the rate was 66%). Separation anxiety disorder was found in one child without SM or social phobia and one child with SM in partial remission, while specific phobias and enuresis nocturna were found in children without SM or social phobia.

When asked about significant negative life events, as assessed by K-SADS, and whether there had been contact with CAMHS or school psychology services during the 5-year follow-up, no negative life events were reported. A majority of the parents (59%) had received some form of consultation/school meetings related to their child’s school functioning. Apart from one child, who had been medicated with SSRIs, and still had SM at follow-up, none had received other kinds of treatment for SM or other anxiety disorders. Most parents reported that they had used what they learned during the treatment period (defocused communication and graded exposure tasks) when they found it appropriate during the follow-up period.

### SM questionnaire data

Table 2 presents mean scores over time. A significant correlation (*p* < 0.001) was found between the SSQ and the SMQ [all SMQ scores > 0.73, apart from the at home-subscale (0.50)]. On the teacher-rated SSQ, there was a small, but significant increase in scores over time (*F*_4,121_ = 24.44, *p* < 0.001), but the higher mean score at T5 was not significantly different from T4, or from T3. SSQ results further indicated a more pronounced increase in speech in younger children. In the model that also included age group as a covariate and a time by age interaction, there was a significant effect of age (*F*_1,30_ = 5.40, *p* = 0.027) and a time by age interaction (a steeper increase of SSQ with time in younger children) (*F*_4,117_ = 2.75, *p* = 0.031), but still significant for time (*F*_4,117_ = 25.84, *p* < 0.001).

**Table 2:** Findings based on teacher and parent questionnaires throughout the study, at baseline (T1), after 3 months of treatment (T2) end of treatment; 6 months (T3), 1 year after end of treatment (T4) and after 5 years (T5)

Informant	Measure	T1 mean (sd)	T2 mean (sd)	T3 mean (sd)	T4 mean (sd)	T5 mean (sd)
Teacher	SSQ	0.54 (0.44)	1.23 (0.93)	1.53 (1.02)	1.54 (0.90)	1.86 (0.77)
Mother	SMQ—school	0.53 (0.43)	1.11 (0.83)	1.45 (0.89)	1.60 (0.90)	2.14 (0.69)
	SMQ—at home	1.65 (0.64)	2.18 (0.47)	2.25 (0.56)	2.32 (0.75)	2.65 (0.37)
	SMQ—in public	0.33 (0.43)	0.70 (0.71)	0.91 (0.69)	1.08 (0.86)	1.90 (0.79)
	SMQ total score	0.86 (0.35)	1.37 (0.53)	1.58 (0.61)	1.81 (0.61)	2.27 (0.55)

*SSQ* school speech questionnaire, *SMQ* selective mutism questionnaire

**Fig. 1:**
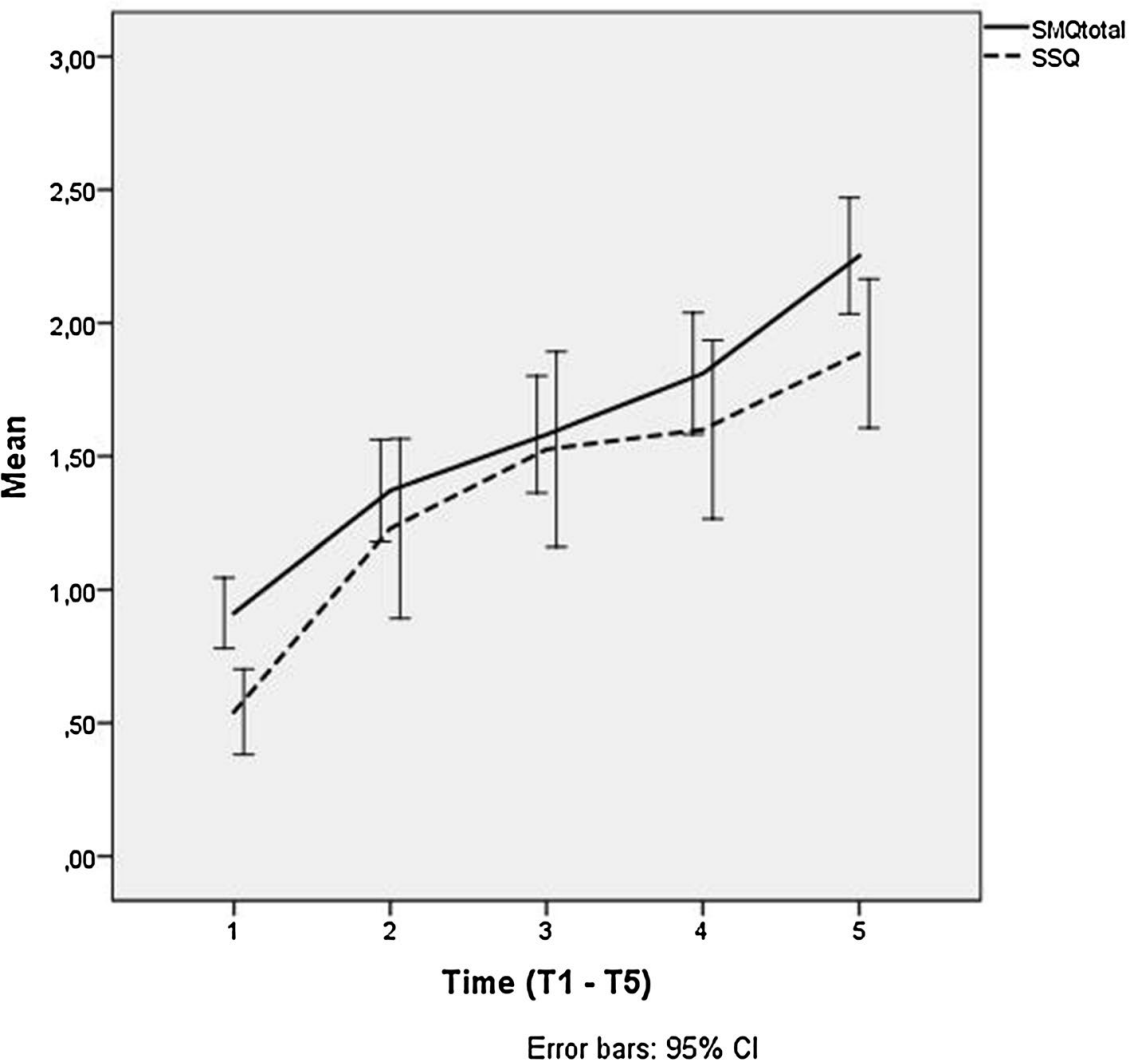
Mean scores on the parent rated SMQ total score and the teacher-rated SSQ over time, at baseline (T1), after 3 months of treatment (T2) end of treatment; 6 months (T3), 1 year after end of treatment (T4) and after 5 years (T5)

The mother-rated SMQ total score showed a significant increase in scores over time (*F*_4,119_ = 28.49, *p* < 0.001), with a significant increase from both T4 to T5, and from T3 to T5 (*p* < 0.001) (Table 2). Again, there was a significant effect of age group (*F*_1,30_ = 12.01, *p* = 0.002), but no significant time by age interaction (*F*_4,115_ = 0.82, *p* = 0.52), see Fig. 1 for an illustration of the change over time on the teacher-rated SSQ and the mother-rated SMQ total score.

Using the SMQ subscale scores, at school, at home and in public, respectively, they all showed significant increases over time, but only the in public-subscale showed a significant increase from T4 to T5 and from T3 to T5 (*p* < 0.001, statistics not shown).

The mean teacher-rated SSQ scores for the three diagnostic groups based on the interview with the mother; No SM, SM in partial remission, and SM were 2.16 (sd = 0.65), 1.47 (sd = 0.25) and 0.83 (sd 0.71), respectively.

A statistically significant omnibus ANOVA F test was found (*F*_2,28_ = 8.89, *p* = 0.001), and the pair of groups was found to be statically significant was SM versus SM in full remission (*p* = 0.001).

### Child rated quality of life

The children reported an overall good quality of life with a mean ILC-LQ_0–28_ score of 22.75 (sd 3.44) and a mean problem score (PR) of 0.89 (sd 1.13), comparable to scores reported for Norwegian schoolchildren (ILC-LQ_0–28_ mean 22.59 (sd 3.88), *p* = 0.82 and PR mean 1.28 (sd 1.60), *p* = 0.18, respectively).

Only one child scored below the reported normative level for ILC-LQ_0–28_ (< 15), and this child did not have SM.

Mean ILC subscale scores are presented in Table 3.

**Table 3:** Quality of Life; ILC ratings by children treated for SM at follow-up (T5)

ILC subscales	Mean (sd)	Range
School	1.82 (0.77)	1–3
Family	1.39 (0.50)	1–2
Other children	1.43 (0.57)	1–3
Alone	2.18 (0.77)	1–4
Physical health	1.71 (0.76)	1–3
Mental health	1.86 (0.65)	1–3
Global life quality	1.50 (0.69)	1–4

*ILC* the inventory of life quality in children and adolescents, with item scores from 1 = very good, 2 = good, 3 = mixed, 4 = bad, 5 = very bad

**Fig. 2:**
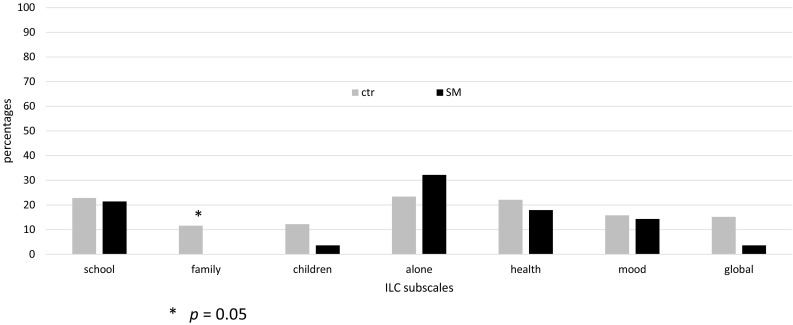
The percentage of problems on the ILC subscales for children in the present study (SM, *n* = 28) versus a sample of Norwegian schoolchildren [44] (Ctr, *n* = 1987)

The percentage of problems on the ILC subscales is in general comparable to data from Norwegian schoolchildren. Scrutinizing the details, the children with SM had significantly less problems at home (*p* = 0.05) and a trend in the direction of better global life quality score (*p* = 0.08), see Fig. 2.

There was no significant difference in child rated quality of life between those without SM (*n* = 20) and those who still had SM or were in partial remission (*n* = 8); 23.4 versus 21.1, *t* = 1.62, *p* = 0.12. When comparing the mean dichotomized ILC problem score whether a problem was considered to be present (1) or not (0), there was a trend, but no significant difference between children without SM (*n* = 20) and children who still had SM or were in partial remission (*n* = 8); 0.65 versus 1.5, *t* = − 1.9, *p* = 0.07.

### Child rated speaking behavior

The mean score on the item measuring difficulties with speaking was 2.29 (sd 0.98) and lies between the scoring alternatives “rather easy” (2) and mixed (3). Half of the children found it “very easy” (*n* = 8) or “rather easy” (*n* = 6) to speak at school and elsewhere outside their home, whereas the other half scored the item as “mixed” (*n* = 12) or rather difficult (*n* = 2). Table 4 lists the number of children in the three groups; no SM (*n* = 20), SM in partial remission (*n* = 5), and SM (*n* = 3) on each of the scores on the 1–5 rating scale.

**Table 4:** Ratings of difficulties with speaking by children treated for SM at follow-up (T5)

Speaking at school/outside home^a^	No SM, (*n* = 20)	SM in remission (*n* = 5)	SM (*n* = 3)
Score 1 = very easy	6, 30%	2 (40%)	0
Score 2 = rather easy	5, 25%	1 (20%)	0
Score 3 = mixed	9, 45%	1 (20%)	2 (67%)
Score 4 = rather difficult	0	1 (20%)	1 (33%)
Score 5 = very difficult	0	0	0

^a^Ratings on a Likert scale, corresponding to a 1–5 range on the ILC

## Discussion

To our knowledge, this is the first prospective follow-up study conducted 5 years after the end of a cognitive behavioral treatment for children with SM in a reasonably large sample (in the context of SM studies). As hypothesized, treatment gains were largely maintained at follow-up (T5).

As shown in Fig. 1, the major improvement took place during the first 3 months of treatment (T1–T2) although the level of treatment intensity remained the same from T2 to T3. We have no obvious explanation for the less steep improvement from T2 to T3. However, our large early effect may be in line with a review of CBT studies for anxiety disorders in youth. The authors say that in the case of most childhood anxiety disorders, treatment responders can expect to be free of their primary diagnosis with a course of treatment that usually last between 12 and 16 weeks [45].

As 70% no longer were diagnosed with SM at the 5-year follow-up, and 17% were in partial remission, this supports provision of CBT treatment for children with SM. We know of no other prospective long-term outcome studies in children with SM to compare with directly. However, our results are good compared to the important CAMELS study on children with anxiety disorders reporting a mean relapse in about half of acute responders when assessed at mean 6 years after randomization. A note of caution is warranted on our definition “SM in partial remission”. This is a tentative definition of the increased occasional use of language in some of the treated children in need of further replication to be a valid description of treatment outcome.

In the first long-term follow-up study based on a larger sample of SM patients, Remschmidt et al. [21] described several psychopathological symptoms as present in young adulthood, including “intermittent mutistic behavior”. Whether the three children that changed status negatively in the present study (one relapse of SM and two diagnosed with SM in partial remission, after having been fluent speakers at the 1-year follow-up) show “intermittent mutistic behavior” or a more persisting mutism, cannot be ascertained in the present study. The negative change could result from several internal or external factors; however, it was not a result of transition into school, as found in our pilot study, because these three children were all school-aged children.

Due to a possibly less entrenched mutism in younger subjects, our finding of a younger age at inclusion to predict more improvement seems plausible. This is also in line with the earliest SM literature suggesting that an early intervention may have been particularly important for those who improved with treatment [8, 46], in studies of the effect of medication in children with SM [7, 15], as well as findings from treatment of children with anxiety disorders in general [34]. As the effect of age at inclusion was not examined in the Bergman study [26], we cannot directly compare our findings on age. However, a retrospective follow-up study including 2/3 of the treated children [27] suggested that a modular treatment of SM (using a component of cognitive training in the form of externalizing the symptoms and cognitive restructuring) was quite beneficial also for older children. Consequently, we cannot rule out that our intervention is more suitable for younger children with SM. One speculation is that for older children with SM, the cognitive component of CBT in the form of active cognitive restructuring could be particularly important, something that was not included in our study.

In line with our previous findings [33], and the study with the longest follow-up time [22], baseline severity of SM, as measured by the parent rated SMQ total score, was a significant negative predictor upon long-term outcome.

Although bilingual children with SM are reported to be overrepresented in several clinical studies [9, 47], and bilingualism is considered a vulnerability factor for SM [9], the present study did not find that bilingualism had a negative impact upon treatment outcome, in line with our previous findings [32, 33]. In general, girls have comprised the majority in recent clinical samples treated for SM [28, 33]. However, a more even gender ratio is also found [27, 33] but whether gender has a predictive value upon treatment outcome, has, as far as we know not been studied. The present long-term-study could not find that gender had a significant impact upon treatment outcome, in line with our findings 1 year after end of treatment [33].

The present study found that having SM in the family (11 of 30 children) was a significant negative predictor of long-term outcome, as only 45% of the children did not have SM, compared to 89% in children without familial SM. This is contrary to our findings 1 year after end of treatment [33] and suggests that familial SM has an increased negative effect on speaking behavior over time. This finding is also in line with the controlled study of long-term outcome of children with SM followed up into young adulthood, showing taciturnity in the family as a negative predictor on psychopathology and symptomatic outcome [22]. Offering booster sessions and long-term follow-up consultations seem particularly relevant for children with a familial SM.

In line with a retrospective follow-up study of children who received treatment for SM [27], we found reduction in comorbid psychiatric disorders. That study postulated that their treatment for SM also decreased the rate of psychiatric comorbidities, including separation anxiety disorder and specific phobia [27]. This is one possible explanation, that CBT for SM has a broader effect over time. One could speculate that being able to speak and being exposed to social situations would reduce especially social phobia. However, it is also likely that a natural development is at play, especially for the elimination disorders and separation anxiety, known for being age dependent [2]. We find it noteworthy that we found a significant reduction in comorbid social phobia as this was found to be a negative predictor of outcome in the CAMS study [34]. However, 23% (*n* = 7) still fulfilled criteria for social phobia, and in line with the retrospective study by Steinhausen et al. [22] with the longest follow-up time (into adulthood), social phobia represented the most prevalent comorbid disorder. This could indicate a need for further interventions to address social phobia in an ongoing manner for children with SM as they become older.

That both the teacher and parent SM questionnaires reported significant improvement over time, and in more settings than just school (at home and in public), supports an overall finding of improvement and could suggests a generalization of effect. For the group as a whole (Table 2), the parent rated SMQ had a mean total score (2.27), indicating that they speak in the range between “Often” and “Always”. However, to define what constitutes a clinically meaningful symptom improvement can be challenging. For the group as a whole (Table 2), we found that the mean teacher reported SSQ scores changed from a level at baseline between “Never” and “Seldom” (0.55) to a level close to “Often” (1.86) at follow-up. We consider this to represent a clinically significant improvement, although not in the suggested range for children without SM (≥ 2.5) [24]. However, ideally, treatment of SM should be continued until there is free speaking in all situations. We do not know whether this would have been achieved by prolonging the same treatment in the present study, or if an active treatment of anxiety had been continued in the local CAMHS. As noted in our previous follow-up studies [32, 33], the main improvement in speaking was found after 3 months of treatment (T2). However, we note that the good outcome found in the retrospective study of 33 of 40 children with SM was achieved after mean 12 months of therapy by one therapist [27], and the lack of active treatment reported by the parents in the present study could support the early findings of general undertreatment of pediatric anxiety disorders [48].

The children in the present study reported good quality of life, comparable to a normative school sample aged 8–16 years [41]. This holds true for both children with and without SM, as the small mean group difference in favor of the children without SM (23.4 versus 21.1) was not statistically significant, and both groups scored above values reported from a Norwegian outpatient sample (*n* = 293, mean score = 20.1) [41]. However, our sample size gave limited statistical power, and there was a tendency on the dichotomized ILC problem score (whether a problem was considered to be present) that children without SM had better quality of life (*p* = 0.07). One could question our use of the normative school sample aged 8–16 years, for boys and girls together (LQ_0–28_ mean = 22.59), as participants in the present study were 8–14 years of age. The manual reports a decline from girls aged 11–12 years and up to the 15–16 year olds [41], leading to a somewhat lower total score for the age range 8–16 years. However, the norms reported for girls aged 8–10 and 11–12 years (a majority in the present study) were 22.92 and 22.85, respectively, relatively similar to our mean score of 22.75.

When rating difficulties with speaking, we find it interesting that 50% of children still do not describe speaking as easy, although most of them speak. One could speculate whether this is related to a personality trait of behavioral inhibition as demonstrated in a study of children with SM [49] and/or taciturnity, in line with the findings of continued problems with communication found in the previously mentioned follow-up study of 45 children with SM [21]. However, whether this subjective anxiety about speaking outside of the home in some children with SM is resistant to change, or whether it would be less prominent with a more intensified CBT, remains to be seen.

The present study demonstrates that the effect of a school-based CBT for SM is largely upheld 5 years after end of treatment in children with SM aged 3–9 years at baseline.

The therapists used our treatment including defocused communication as a general treatment principle, and a home and school-based intervention with gradual exposure to the feared situations in which speech is expected. We find it particularly promising that we could observe a significant effect, when our CAMHS therapists were not experts in SM or CBT. Our study is different from the RCT study using CBT trained therapists, working at one clinic under direct guidance by the principal investigator [26], or the two uncontrolled studies where all children were treated by one particularly dedicated therapist [27, 28] recruiting patients from private practice [28]. In spite of a considerably longer treatment time, and a relatively large drop-out [27], their results are very promising. Although teacher-rated severity was not included in the two uncontrolled studies, their parent rated SMQ-School subscale scores do not suggest that these children had a less severe SM (potentially more easy to treat) than children in our study. For older children with SM, how to better utilize available school resources could be investigated further. One could also speculate that a more active cognitive restructuring component, included in some studies [27, 28] could be essential for the older children with SM, especially.

Based on our inclusion criteria, specifying that the children should not talk to adults/teachers, one could suspect that our sample is selected, including more severely impaired children than other treatment studies. However, when comparing baseline severity, as rated by the parent rated SMQ-School subscale with recent studies, the scores are very similar. Our score is 0.53, the two uncontrolled studies report 0.52 and 0.53 [27, 28] and the controlled Bergman study report 0.40 on the SMQ-school subscale and 0.66 on the teacher-rated SSQ [26]. This suggests that our sample is comparable to other recent and important treatment studies.

Future research is needed to ascertain whether there are particularly important treatment components. The elements of our treatment are quite similar to previous controlled [26] and uncontrolled [27, 28] treatment studies for children with SM. The central elements are a very close cooperation with the school, underlining both child- and parent-engagement, and the behavioral component of gradual exposure to the feared stimulus (e.g., speaking) is emphasized.

Although the recent treatment studies with larger samples of children with SM have used CBT with a weight on the behavioral (exposure) component, a note of caution is that the superiority of CBT over any other intervention for SM still has to be supported by other study designs. Likewise, studies analyzing the minority of less satisfactory courses or treatment refractory cases still lie ahead.

### Limitations

Sample size was limited. Questionnaire data (not diagnoses) were available at the end of treatment (6 months, T3). We also have limited detailed knowledge about their follow-up at school and from CAMHS, apart from the fact that no other psychosocial treatment was given. The loss of two children at T5, who had SM at T4 could perhaps have given larger differences in measures such as quality of life.

## Conclusions

This is the first prospective follow-up study conducted 5 years after the end of a cognitive behavioral treatment for children with SM, in a reasonably large sample (e.g., in the context of SM). Clinical gains were largely maintained at follow-up, as rated by a both a child psychiatrist, teachers, and parents. A significant reduction of comorbid anxiety disorders was found, and the children reported good quality of life. However, half of the children still described it as somewhat challenging to talk at school/outside home, although the majority did speak. Several children had persistent SM symptoms, speaking to the need for the study of additional interventions to help such individuals.
